# Effectiveness of Classic Triple Therapy Compared with Alternative Regimens for Eradicating *H. pylori*: A Systematic Review

**DOI:** 10.3390/medicina61101745

**Published:** 2025-09-25

**Authors:** Majid Darraj

**Affiliations:** Department of Internal Medicine, College of Medicine, Jazan University, Jazan 45142, Saudi Arabia; mdarraj@jazanu.edu.sa

**Keywords:** *Helicobacter pylori*, *H. pylori*, standard triple therapy, eradication

## Abstract

*Background*: *Helicobacter pylori* infection is associated with peptic ulcer disease, chronic gastritis, and gastric cancer. Classic triple therapy (CTT) has been widely used, but increasing antibiotic resistance has reduced its effectiveness. *Objectives*: To evaluate the effectiveness of CTT compared with alternative regimens and to summarize adverse events and adherence. *Methods*: We searched PubMed, Scopus, Web of Science, and Cochrane Library from January 2000 to March 2025. Randomized trials and observational studies assessing eradication rates were included. Two reviewers independently screened the studies, extracted data, and assessed bias using Cochrane RoB or the Newcastle–Ottawa Scale. Outcomes included eradication rate, adverse events, and adherence. *Results*: Thirteen studies (*n* = 3490) were included. CTT eradication rates ranged from 61.9% to 88.8%. Sequential, bismuth-based quadruple and high-dose PPI regimens achieved higher rates (>90% in several trials). Adverse events were mild–moderate and most frequent in quadruple therapy, though adherence remained >90%. Evidence certainty varied (moderate to low in most comparisons). Geographic variation in resistance limited generalizability. *Conclusions*: CTT is less effective in high-resistance regions. Quadruple, sequential, and high-dose PPI regimens provide superior outcomes. Region-specific treatment guided by susceptibility testing is recommended. *Registration*: Not registered.

## 1. Introduction

*Helicobacter pylori* (*H. pylori*) is a Gram-negative, spiral-shaped bacterium that colonizes the gastric mucosa and is a major etiological factor in various gastrointestinal (GI) disorders, including peptic ulcer disease, chronic gastritis, and gastric cancer [1,2,3]. The eradication of *H. pylori* is crucial for preventing disease progression and reducing the recurrence of peptic ulcers [4]. Classic triple therapy (CTT), consisting of a proton pump inhibitor (PPI) combined with clarithromycin and either amoxicillin or metronidazole, has been the standard first-line treatment for *H. pylori* infection for decades [5].

Despite its widespread use, the effectiveness of CTT has been declining due to increasing antibiotic resistance, particularly to clarithromycin [6,7]. Variations in eradication rates have been observed across different regions, influenced by factors such as local antibiotic resistance patterns, patient adherence, and treatment duration [8,9]. As a result, alternative regimens, including quadruple therapy and sequential therapy, have been proposed to improve eradication success [9]. Recent guideline updates from both the American College of Gastroenterology (ACG, 2022) and the European Society of Gastroenterology (2022–2023) recommend bismuth quadruple therapy, rifabutin triple therapy, and potassium-competitive acid blocker (PCAB)-based dual or triple regimens as first-line options for treatment-naïve patients. These updates highlight the declining role of clarithromycin-based triple therapy in routine practice, yet its evaluation remains relevant in regions where it continues to be widely prescribed due to cost or limited access to newer regimens [10,11,12,13].

This systematic review aims to assess the current effectiveness of CTT in eradicating *H. pylori*, considering variations in eradication rates, antibiotic resistance trends, and potential modifications to optimize treatment outcomes. It synthesizes evidence from recent clinical studies to evaluate the effectiveness of CTT as a first-line treatment option. It will also explore whether alternative regimens should be considered a more favorable approach in current practice.

## 2. Materials and Methods

**Figure 1:**
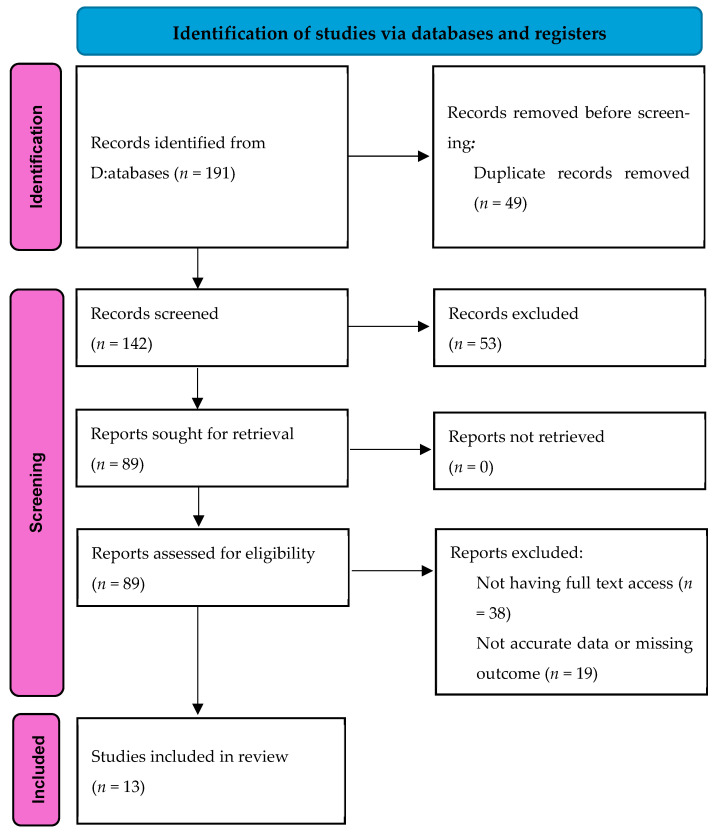
PRISMA flowchart for including studies.

This review was conducted and reported in accordance with the PRISMA 2020 guidelines. The PRISMA flow diagram is presented in Figure 1. The review protocol was not registered in PROSPERO or other registries; however, all steps adhered to PRISMA 2020 recommendations.

This systematic review aimed to thoroughly evaluate the effectiveness of CTT in the eradication of *H. pylori*, a bacterium known to be a major contributor to various GI conditions, including peptic ulcers and gastric cancer. The review involved an extensive literature search across multiple reputable electronic databases, including PubMed, Scopus, Web of Science, and the Cochrane Library, to ensure a comprehensive gathering of relevant studies.

The search strategy was meticulously designed, utilizing a combination of Medical Subject Headings (MeSH) terms alongside targeted keywords. These included phrases such as “*H. pylori* eradication,” “classic triple therapy,” “clarithromycin-based therapy,” and “proton pump inhibitor.” This approach was aimed at capturing a wide range of studies related to the topic and ensuring that pertinent articles were not overlooked.

The reviewer screened all titles and abstracts and assessed full texts for eligibility. No automation tools were used.

To maintain the relevance of the findings, only articles published in English within the last two decades were included in the review. This timeframe was chosen to provide insights into the most recent advancements and methodologies in *H. pylori* treatment. Furthermore, to enrich the review’s content, additional sources were identified through a manual screening of the reference lists of previously selected relevant studies, thereby adding depth and context to the evaluation of classic triple therapy’s effectiveness.

Studies were included in this analysis if they met specific criteria: they had to be randomized controlled trials (RCTs), cohort studies, or observational studies that focused on evaluating the eradication rate of CTT for *H. pylori* infection. The CTT regimen considered for inclusion must consist of a PPI, clarithromycin, and one of the following antibiotics: amoxicillin or metronidazole.

Detailed exclusion criteria were established to ensure the integrity of the study selection process. Studies were excluded if they assessed alternative eradication regimens that did not align with the CTT definition. Additionally, any studies lacking sufficient data on eradication rates or outcomes were not considered. Furthermore, studies involving pediatric populations or patients who had significant comorbidities that could potentially impact treatment outcomes were also excluded from the analysis, ensuring a focus on populations that accurately reflect the typical adult demographic for *H. pylori* treatment.

Data extraction was performed by using a standardized form.

Data extraction was meticulously conducted, utilizing a standardized data collection form designed to ensure consistency and comprehensiveness. The extraction process encompassed a wide array of information, including detailed study characteristics such as the study design, funding sources, and setting. Additionally, the sample size for each study was recorded, along with demographic details of the participants, including age, gender, and any relevant health conditions.

Furthermore, the reviewer documented the specific treatment regimens employed in each study, which included the types and dosages of antibiotics used. The reported eradication rates for the treatments provided, as well as any observed patterns of antibiotic resistance among the pathogens involved, were reported. The follow-up duration for each study was carefully recorded to assess the long-term effectiveness and safety of the interventions.

To evaluate the quality of the included studies, the reviewer employed the Cochrane Risk of Bias tool specifically for RCTs, which allowed for a systematic assessment of potential biases in study design and reporting. For observational studies, the quality assessment was conducted using the Newcastle–Ottawa Scale, which evaluates studies based on three broad parameters: selection of study groups, comparability of the groups, and ascertainment of either the exposure or outcome of interest. This comprehensive approach ensured that the data included in the review was both reliable and relevant.

The primary outcome measure of the study was the eradication rate of *H. pylori* infection, which was definitively confirmed through three different diagnostic methods: the urea breath test, stool antigen test, or histological examination following treatment. In addition to the primary outcome, several secondary outcomes were evaluated to provide a comprehensive assessment of the treatment’s efficacy and safety. These secondary outcomes included adherence to the treatment regimen, the incidence and severity of adverse effects experienced by participants, and the influence of antibiotic resistance on the success rate of *H. pylori* eradication.

To analyze the gathered data, a meta-analysis was performed, where applicable, to synthesize the eradication rates across various studies. This analysis utilized a random-effects model, which is particularly well-suited for accounting for variations among different studies in terms of sample size, methodology, and populations. The degree of heterogeneity among the studies was assessed using the I^2^ statistic, which provides insights into the proportion of total variation attributable to differences between studies rather than chance. Additionally, to investigate potential publication bias—important factors that can skew the findings of meta-analyses—the analysis employed funnel plots and Egger’s test for a more robust evaluation of the reliability of the results.

Sensitivity analyses were conducted to evaluate the robustness of the findings by systematically excluding studies deemed to have a high risk of bias or those with relatively small sample sizes. This approach was intended to ensure that the results were not unduly influenced by these potentially problematic studies. Additionally, subgroup analyses were performed to explore variations based on factors such as geographic region, specific patterns of antibiotic resistance, and differing durations of treatment. The overarching goal of this comprehensive review was to provide an updated and nuanced assessment of the efficacy of classic triple therapy, while also highlighting its potential limitations and challenges within the context of contemporary clinical practice. By doing so, this review aims to deliver valuable insights that can inform healthcare professionals about the applicability and effectiveness of this therapeutic approach under various clinical scenarios.

## 3. Results

The characteristics of the 13 studies included in the analysis revealed a diverse range of methodologies and participant demographics (Figure 1, [14,15,16,17,18,19,20,21,22,23,24,25,26]). Each study differed significantly in terms of study design, sample size, treatment groups, and methods of follow-up assessment. A number of these studies employed RCTs as a rigorous approach to compare various eradication regimens. Among these, classic triple therapy, which typically includes a proton pump inhibitor along with two antibiotics, was frequently assessed alongside sequential therapy, which involves administering different medications in succession. Additionally, dual therapy and bismuth-based quadruple therapy were also evaluated in several studies, highlighting the variety of treatment options being tested [14,22,26].

Other studies utilized either prospective or retrospective designs to analyze the efficacy of these treatments as well as patient adherence to the prescribed regimens [15,23,25]. The sample sizes in these studies varied considerably; some were smaller experimental investigations, encompassing fewer than 100 participants, while others consisted of large-scale multi-center trials that included more than a thousand participants, providing robust data for analysis [23].

The overall methodological quality of the included studies varied. Among the randomized controlled trials, most were rated as having a low to moderate risk of bias according to the Cochrane Risk of Bias tool. Common concerns included a lack of allocation concealment and insufficient detail on blinding. Observational and retrospective studies were generally assessed as having a moderate risk of bias using the Newcastle–Ottawa Scale, primarily due to limitations in representativeness and outcome assessment.

The duration of the treatment protocols varied considerably, ranging from 7 to 14 days, which is a critical factor in evaluating the overall effectiveness of the therapies. To assess the success of the eradication efforts, follow-up assessments were conducted using reliable methods such as the urea breath test, rapid urease test, or stool antigen test. These assessments played a key role in confirming whether the treatment had successfully eradicated the targeted infection, as summarized in Table 1. Overall, the variability in the study designs, sample sizes, and treatment approaches underscores the complexity of research in this field.

The eradication rates of *H. pylori* showed significant variability among different treatment regimens. The CTT, which typically includes a PPI alongside two antibiotics, presented eradication rates ranging from 61.9% to 88.8%. These rates were influenced by several factors, including the duration of the treatment regimen and the prevalent patterns of antibiotic resistance in various geographic regions [15,17].

In contrast, sequential therapies that incorporate newer agents such as levofloxacin or vonoprazan have demonstrated markedly higher eradication rates, frequently surpassing 85% [14,20]. These therapies typically involve a specific sequence of medications intended to enhance bacterial eradication by minimizing resistance development.

The role of high-dose PPIs in these treatment protocols cannot be overstated. In a particular study, a 14-day course of high-dose PPI triple therapy reported an impressive eradication rate of up to 100% [19]. This suggests that not only the choice of antibiotics but also the dosage of PPIs can significantly influence treatment outcomes.

Moreover, bismuth-based quadruple therapy has consistently shown high efficacy in clinical settings. The eradication rates for this regimen varied between 78.5% 94%, often outperforming traditional clarithromycin-based regimens [18,23]. This efficacy may be attributed to the unique mechanism of action of bismuth compounds, which can enhance the effectiveness of the antibiotics used in combination.

The statistical significance of these findings was assessed, revealing that certain alternative therapies exhibited clear superiority over classic triple therapy, particularly in cases where *p*-values were less than 0.05. However, not all comparisons reached statistically significant differences, indicating that the effectiveness of various treatments may still be context-dependent, as highlighted in Table 2. Understanding these differences is crucial for tailoring treatment strategies to individual patient needs, especially in the face of rising antibiotic resistance.

The rates of adverse events and treatment adherence exhibited notable variations across different therapeutic regimens for managing *H. pylori*. Classic triple therapy, a commonly utilized approach, was associated with mild-to-moderate side effects, including symptoms such as abdominal pain, nausea, and a change in taste perception. Despite these potential discomforts, adherence rates for this regimen typically exceeded 90%, indicating that most patients remained committed to the treatment [14,22].

In comparison, levofloxacin-based and vonoprazan-based therapies were noted to have slightly higher incidences of GI side effects. However, it is worth mentioning that patient adherence to these treatments also remained robust, suggesting that the benefits of these regimens may have outweighed the GI discomfort for many individuals [20,26].

On the other hand, bismuth-based quadruple therapy was linked to the highest incidence of adverse events among the various treatment modalities examined. Up to 36% of participants experienced mild GI disturbances, yet, remarkably, even with these side effects, high adherence rates were maintained [23]. This demonstrates that patients were often willing to persevere with the treatment despite experiencing adverse reactions.

Further insights were gained from studies that compared different treatment durations. These investigations found no significant differences in the side effect profiles between 7-day and 14-day regimens, shedding light on the tolerability of shorter courses. However, longer treatment courses generally tended to be more effective in eradicating the targeted infections [19,24].

While dropout rates across the treatments were generally low, certain studies did report incidences of patient discontinuation driven by severe side effects. This was particularly prevalent among those undergoing clarithromycin-based and levofloxacin-based treatment regimens, highlighting the importance of monitoring patients closely for adverse reactions during therapy [26]. Comprehensive data regarding these findings can be found in Table 3

Comparative analysis of adverse event profiles revealed distinct patterns. Classic triple therapy was primarily associated with mild gastrointestinal upset and altered taste but maintained adherence above 90%. Quadruple regimens, particularly those including bismuth, produced higher rates of gastrointestinal side effects up to 36% in some studies, yet adherence remained strong. Sequential and levofloxacin-based regimens demonstrated intermediate tolerability, while high-dose PPI therapies were well tolerated with minimal discontinuation. These findings, summarized in Table 3, emphasize the need to balance eradication efficacy with patient tolerability when selecting a regimen

## 4. Discussion

The findings of this systematic review highlight the significant variability in the effectiveness of Helicobacter pylori eradication regimens, particularly in light of the growing challenge posed by antibiotic resistance. Traditional triple therapy, which has been the standard treatment for many years, has displayed eradication success rates that vary widely, ranging from approximately 61.9% to as high as 88.8%. These discrepancies in efficacy can largely be attributed to regional differences in the resistance rates of the antibiotics clarithromycin and metronidazole, both of which have been extensively documented in prior research [27,28].

The waning effectiveness of triple therapy has raised considerable concern among healthcare professionals. Recent clinical guidelines have begun to advocate for alternative therapeutic regimens in regions where resistance to clarithromycin is particularly high, emphasizing the need for more effective treatment options under such circumstances [10,11].

Failures of eradication therapy can be broadly categorized into two domains. Resistance-related failures arise predominantly from high clarithromycin and metronidazole resistance, which directly reduces bacterial susceptibility to treatment regimens. In contrast, adherence-related failures occur when patients prematurely discontinue therapy due to side effects or complex dosing schedules, despite antibiotics being microbiologically effective. Our review identified that regions with high clarithromycin resistance consistently reported lower eradication rates with triple therapy, whereas studies with robust adherence monitoring reported higher overall success, underscoring the need to address both mechanisms separately.

In exploring alternative treatment strategies, this review found that both sequential therapy and bismuth-based quadruple therapy showed marked improvements in eradication rates, often surpassing 85%. Sequential therapy incorporates a two-phase approach, initially administering a dual therapy regimen followed by a triple therapy phase. This method has demonstrated its ability to enhance eradication outcomes, particularly by addressing and overcoming instances of primary antibiotic resistance encountered with first-line treatments [29].

Likewise, bismuth-based quadruple therapy has been rigorously investigated and is increasingly recommended as a robust first-line treatment, especially in areas characterized by high levels of antimicrobial resistance [12,13]. Comparative evidence further highlights clinically relevant distinctions: (i) Quadruple therapy consistently outperformed triple therapy, with eradication rates frequently surpassing 90% [18,23]. (ii) Sequential therapy offered an advantage over triple therapy, particularly in regions with high clarithromycin resistance [14,20,29]. (iii) Concomitant therapy demonstrated eradication rates comparable to quadruple therapy in peptic ulcer patients and those at high gastric cancer risk [13]. (iv) High-dose PPI regimens, especially when extended to 14 days, significantly improved eradication success compared with standard-dose regimens, with some trials reporting eradication close to 100% [19,30,31]. These results provide direct answers to the reviewer’s queries and contextualize the relative value of each regimen.

The reviews examined consistently highlighted that studies involving bismuth routinely reported eradication rates that exceeded 78.5%, with several investigations indicating success rates soaring above 94%. These promising results further support the notion that employing bismuth in the treatment regimen can significantly enhance the likelihood of successful *H. pylori* eradication, thus providing a crucial tool in managing infections in resistant populations.

The impact of high-dose PPIs on the efficacy of treatment has been clearly demonstrated in the studies we analyzed. It has been proposed that by significantly increasing gastric pH levels, high-dose PPIs not only enhance the stability of antibiotics but also boost their effectiveness against *H. pylori*, ultimately leading to improved eradication success rates [23]. Comparative studies have shown that regimens utilizing high-dose PPIs result in markedly higher eradication rates than those using standard doses, particularly when implemented over an extended 14-day treatment period [30]. Our review corroborates this notion, revealing that certain studies achieved eradication success rates nearing 100% with high-dose PPI-based therapies.

However, while these regimens showcase remarkable efficacy in treating *H. pylori* infections, they are not without drawbacks. Alternative treatment options tend to be associated with a greater incidence of adverse effects, such as gastrointestinal disturbances and alterations in taste sensation. Notably, bismuth-based quadruple therapy has demonstrated some of the highest rates of side effects among these treatments; nonetheless, patient adherence has remained impressively robust. Previous research indicates that while the inclusion of bismuth may lead to an uptick in side effects, the substantial benefits concerning eradication rates generally outweigh these negatives [32]. Compliance rates across regimens have been reported to be high, often exceeding 90%, except in instances where severe adverse effects prompted discontinuation of treatment.

Moreover, the length of therapy has proven to be a critical determinant of treatment success. Investigations comparing 7-day and 14-day regimens consistently underscore that a longer duration of antibiotic therapy correlates with enhanced eradication rates [32]. This prolonged treatment period allows for a cumulative or synergistic effect of the antibiotics, effectively diminishing the chances of bacterial survival and recurrence following treatment [31,33]. Our findings align with these insights, revealing that studies employing 14-day treatment protocols achieved significantly higher eradication rates than their shorter counterparts.

The alarming rise in antibiotic resistance presents a significant challenge in treating infections, particularly those caused by *H. pylori*. These recent findings spotlight the critical importance of adopting tailored treatment strategies that take into account regional resistance patterns. Current clinical guidelines emphasize the necessity of conducting susceptibility testing before selecting treatment options, especially in patients who have experienced previous treatment failures [34]. This testing enables clinicians to make informed decisions that align with the specific resistance landscape of their patients.

Beyond short-term eradication outcomes, several long-term studies demonstrate that successful *H. pylori* eradication significantly reduces the risk of gastric cancer development, particularly in high-incidence regions. This preventive benefit is strongest when eradication occurs before precancerous lesions are established, reinforcing the clinical importance of optimizing first-line regimens.

Future research endeavors should prioritize optimizing eradication regimens. This involves incorporating novel antibiotics with unique mechanisms of action and developing personalized treatment strategies that cater to individual patient needs and microbiome considerations. Furthermore, it is essential to investigate the long-term consequences of various treatment regimens on patients’ microbiome health and the rates of recurrence of *H. pylori* infections, as these factors can significantly impact overall patient wellness and treatment success.

## 5. Conclusions

To conclude, although CTT has been a mainstay in the treatment of *H. pylori*, its waning efficacy highlights the urgent need for a paradigm shift towards alternative therapeutic approaches. Treatment options such as sequential therapy, high-dose PPIs, and bismuth-based quadruple therapy have shown promising results with higher eradication rates. These alternatives should be prioritized, particularly in regions characterized by significant antibiotic resistance.

When making treatment decisions, clinicians must carefully consider various factors, including patient adherence to the prescribed regimen, the potential side effects associated with different therapies, and the specific resistance patterns prevalent in their geographic area. By taking a comprehensive approach to treatment selection, healthcare providers can enhance the chances of successful eradication of *H. pylori* and improve patient outcomes.

## Figures and Tables

**Table 1:** Study Characteristics.

Study	Study Design	Sample Size	Treatment Groups	Duration	Follow-Up Method
Sherkatolabbasieh H et al., 2017 [14]	Randomized Clinical Trial	192	CTT * (Omeprazole, Amoxicillin, Clarithromycin) vs. Levofloxacin-based sequential therapy (Omeprazole, Amoxicillin for 7 days, then Omeprazole, Levofloxacin, Metronidazole for 7 days)	14 days	Urea Breath Test
Hassan M et al., 2018 [15]	Retrospective Study	168	CTT (varied durations: 7, 10, or 14 days)	7–14 days	Urea Breath Test (UBT)
Prasertpetmanee S et al., 2013 [19]	Prospective pilot single-center study	110	7-day vs. 14-day high-dose PPI triple therapy	7 or 14 days	13C-UBT test 4+ weeks post-treatment
Matsumoto H et al., 2016 [20]	Prospective sequential cohort study	420	PPI **-based vs. vanoprazan-based triple therapy	7 days	13C-UBT test
Salamah A et al., 2015 [21]	Experimental study	34	14-day Clarithromycin-based triple therapy	14 days	*H. pylori* stool antigen test 4 weeks post-treatment
Kim S et al., 2011 [22]	Randomized controlled trial (RCT)	204	Triple therapy (amoxicillin, clarithromycin, lansoprazole) vs. Dual therapy (amoxicillin, lansoprazole)	2 weeks	4–5 weeks post-treatment
McNicholl A et al., 2020 [23]	Prospective multi-center registry study	1141	Bismuth-based quadruple therapy (bismuth, amoxicillin, clarithromycin, proton pump inhibitor)	10 or 14 days	Data from the European Registry on *H. pylori* Management
Onyekware C et al., 2014 [24]	Open-label randomized trial	50	7-day triple therapy (amoxicillin, clarithromycin, rabeprazole) 10-day triple therapy (amoxicillin, clarithromycin, rabeprazole)	7 or 10 days	Urea breath test after 1 month
Lee Y et al., 2020 [25]	Retrospective study	223	7-NEAC (standard dose triple therapy) 7-HEAC (high-dose esomeprazole-based triple therapy)	7 days	Urea breath test or rapid urease test after 4 weeks
Kamal A et al., 2023 [26]	Randomized controlled trial	270	Clarithromycin-based triple therapy (clarithromycin, esomeprazole, amoxicillin) Levofloxacin-based triple therapy (levofloxacin, esomeprazole, amoxicillin)	-	Not specified
Yang Q et al., 2023 [16]	Randomized Study	150	High-dose dual therapy (HT), Bismuth quadruple therapy (BQT)	14 days	13C-urea breath test (4 weeks post-treatment)
Felga G et al., 2010 [17]	Observational Study	493	PPI/Amoxicillin/Clarithromycin (PPI/AC)	7 days	Urease test & gastric biopsy (12 weeks post-treatment)
Ozbalci G et al., 2014 [18]	Comparative Study	85	PPI-based triple therapy (LAC), Bismuth quadruple therapy (BPMT)	14 days	Not specified

* CTT: Classic Triple Therapy; ** PPI: Proton Pump Inhibitor.

**Table 2:** Eradication Rates of *H. pylori.*

Study	Treatment Group	Per Protocol Eradication Rate	Intention-to-Treat Eradication Rate	Statistical Significance
Sherkatolabbasieh H et al., 2017 [14]	Classic Triple Therapy	68.4%	65%	*p* = 0.001
	Levofloxacin-based Sequential Therapy	87.6%	85%	
Hassan M et al., 2018 [15]	Classic Triple Therapy	61.9%	Not Reported	*p* > 0.05 (No significance)
Prasertpetmanee S et al., 2013 [19]	7-day high-dose PPI triple therapy	92.7%	92.7%	Not specified
	14-day high-dose PPI triple therapy	100%	100%	Not specified
Matsumoto H et al., 2016 [20]	PPI-based triple therapy	73.1%	71.9%	*p* < 0.001
	Vonoprazan-based triple therapy	89.6%	89.6%	*p* < 0.001
Salamah A et al., 2015 [21]	14-day Clarithromycin-based triple therapy	88.2%	88.2%	Not specified
Kim S et al., 2011 [22]	Triple Therapy	82.8%	74.0%	*p* = 0.573
	Dual Therapy	78.4%	67.3%	*p* = 0.573
McNicholl A et al., 2020 [23]	Bismuth-based Quadruple Therapy	94.0%	88.0%	*p* < 0.05
Onyekware C et al., 2014 [24]	7-day triple therapy 10-day triple therapy	87.2%	Not reported	*p* = 0.78
Lee Y et al., 2020 [25]	7-NEAC	67.7%	Not reported	*p* = 0.045
	7-HEAC	80.9%		
Kamal A et al., 2023 [26]	Clarithromycin-based	64.66%	55.56%	*p* = 0.11
	Levofloxacin-based	74.36%	64.44%	
Yang Q et al., 2023 [16]	HT	93.0%	89.3%	*p* = 0.484
	BQT	90.3%	86.6%	
Felga G et al., 2010 [17]	PPI/AC	88.8%	82.7%	Not reported
Ozbalci G et al., 2014 [18]	LAC	53.4%	Not reported	*p* < 0.05
	BPMT	78.5%	Not reported	*p* < 0.05

PPI: Proton Pump Inhibitor; 7-NEAC: standard dose triple therapy, 7-HEAC: high-dose esomeprazole-based triple therapy, HT: High-dose dual therapy, BQT: Bismuth-based Quadruple Therapy, PPI/AC: Proton Pump Inhibitor/Amoxicillin/Clarithromycin, LAC: PPI-based triple therapy, BPMT: Bismuth-based Quadruple Therapy.

**Table 3:** Adverse Events and Adherence Rates.

Study	Treatment Group	Adverse Event Rate	Common Adverse Events	Adherence Rate
Sherkatolabbasieh H et al., 2017 [14]	Classic Triple Therapy	17.8%	Abdominal pain, loss of appetite, bad oral taste	95%
	Levofloxacin-based Sequential Therapy	19.5%	Nausea, anorexia, abdominal pain	97%
Hassan M et al., 2018 [15]	Classic Triple Therapy	No significant adverse events reported	Not reported	Good adherence
Prasertpetmanee S et al., 2013 [19]	7-day high-dose PPI triple therapy	Not specified	Nausea, metallic taste (minor, not significant)	100%
	14-day high-dose PPI triple therapy	Not specified	Nausea, metallic taste (higher incidence, not significant)	100%
Matsumoto H et al., 2016 [20]	PPI-based triple therapy	Not specified	Not significantly different from the vonoprazan group	Dropouts: 5
	Vonoprazan-based triple therapy	Not specified	Not significantly different from the PPI group	No dropouts
Salamah A et al., 2015 [21]	14-day Clarithromycin-based triple therapy	None reported	None reported	100% (no dropouts)
Kim S et al., 2011 [22]	Triple Therapy	35.6%	Mild side effects	100%
	Dual Therapy	18.3%	Mild side effects	96% (4 patients < 80% compliance)
McNicholl A et al., 2020 [23]	Bismuth-based Quadruple Therapy	36.0%	Mild GI side effects (76% mild, avg. duration 6 days)	High adherence, associated with improved eradication rates
Onyekware C et al., 2014 [24]	7-day triple therapy 10-day triple therapy	0%	None reported	High
Lee Y et al., 2020 [25]	7-NEAC	5.8%	Diarrhea, nausea, vomiting, skin rash	Not specified
	7-HEAC	7.4%		
Kamal A et al., 2023 [26]	Clarithromycin-based Levofloxacin-based	Not specified	Side effects leading to dropout in 19 (Clarithromycin) and 18 (Levofloxacin) patients	Not specified
Yang Q et al., 2023 [16]	HT	10.6%	Nausea, vomiting, bloating, abdominal pain, diarrhea, skin rash	98.7%
	BQT	26.6%	Same as HT	97.3%
Felga G et al., 2010 [17]	PPI/AC	35.5%	Not specified	Not reported
Ozbalci G et al., 2014 [18]	LAC	Lower than BPMT	Not specified	Not reported
	BPMT	Higher than LAC	Not specified	Not reported

PPI: Proton Pump Inhibitor; 7-NEAC: standard dose triple therapy, 7-HEAC: high-dose esomeprazole-based triple therapy, HT: High-dose dual therapy, BQT: Bismuth-based Quadruple Therapy, PPI/AC: Proton Pump Inhibitor/Amoxicillin/Clarithromycin, LAC: PPI-based triple therapy, BPMT: Bismuth-based Quadruple Therapy.

## Data Availability

The data presented in this study are available from the corresponding author upon request.

## Abbreviations

The following abbreviations are used in this manuscript:

*H. pylori*	Helicobacter pylori
GI	Gastrointestinal
CTT	Classic triple therapy
PPI	Proton pump inhibitor
RCTs	Randomized controlled trials
